# Repurposing of archived CO1 sequence data reveals unusually high genetic structure between North American and European zebra mussels (*Dreissena polymorpha*)

**DOI:** 10.1080/23802359.2017.1407713

**Published:** 2017-11-25

**Authors:** Andrew A. David, Kendall Gardner

**Affiliations:** Department of Biology, Clarkson University, Potsdam, NY, USA

**Keywords:** *Dreissena polymorpha*, Laurentian, big data, invasion

## Abstract

The invasion of the zebra mussel, *Dreissena polymorpha* in the Great Lakes of North America is regarded as one of the most catastrophic ecological events in recent history. Previous studies showed a close kinship between European zebra mussels and their invasive cohorts in the Great Lakes. In this study, we repurposed and reanalyzed archived CO1 sequence data from Lake Superior and multiple sites in Europe that were collected between 1991 and 2011 to illustrate an interesting pattern of genetic isolation that was overlooked in previous studies. The results showed extreme genetic isolation of Lake Superior zebra mussels as evident by high ϕ_ST_ values and strong geographic patterning of Lake Superior haplotypes.

## Introduction

The zebra mussel, *Dreissena polymorpha*, is the most invasive freshwater mollusc in the United States, where its economic impact is estimated in the billions of dollars (Strayer 2008). The mussel is native to Europe, specifically the waters of the Ponto-Caspian Basin. This region was hypothesized as the source for the first North American cohorts of *D. polymorpha*, which arrived via transportation in ballast water and quickly became established in Lake St. Clair in the mid-1980s (May et al. 2006). Since then, the species has spread to all of the Great Lakes and has transformed their chemistry leading to the extirpation of many native species while also facilitating invasion of other freshwater exotics (Simberloff and Von Holle 1999).

Population genetics is a critical tool for understanding crucial aspects of the invasion process due to the difficulty in tracking individuals in vast lake and ocean basins (Le Roux and Wieczorek 2009). Understanding the dispersal and connectivity patterns of invasive populations can help track the spread of invasive species and more importantly, help conservationists detect isolated point sources which could be targeted for eradication (Robertson and Gemmell 2004). However, a population genetic study merely gives a ‘freeze-frame’ of the invasion process which can be extremely dynamic due to stochastic factors such as changes in vector strength, genetic drift and transient dispersal barriers. To address this, a temporal approach to population genetics has been suggested (Skoglund et al. 2014). Unfortunately, acquiring temporal samples across a species’ native and introduced range is time consuming and not always feasible due to logistics and limited funding. Alternative and often underutilized resources that may solve this problem are DNA sequence databases.

As the cost of sequencing continues to plummet, sequence databases are growing at an exponential rate (Pope et al. 2015). While these databases are important for the reproducibility of published research, they have remained grossly underutilized in the field of invasion genetics, despite the fact that they possess a wealth of spatio-temporal data (Denk 2017). In this study, we attempted to repurpose archived mtDNA sequences from public databases collected across multiple years to investigate connectivity patterns of *D. polymorpha* from Europe and North America.

## Methods and results

**Figure 1.**
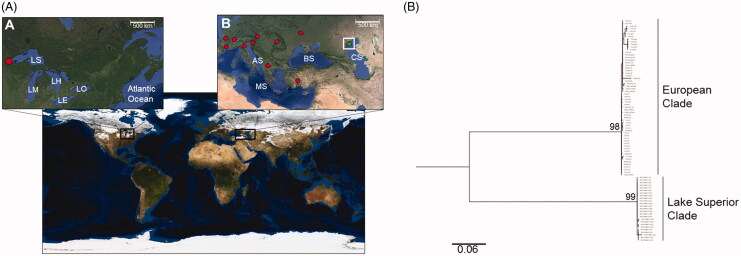
(A) Map showing broad sampling localities of archived CO1 sequences of *Dreissena polymorpha* from North America and Europe. LS: Lake Superior; LM: Lake Michigan; LH: Lake Huron; LE: Lake Erie; LO: Lake Ontario; CS: Caspian Sea (native range); BS: Black Sea; MS: Mediterranean Sea; AS: Adriatic Sea. (B) Bayesian tree obtain from the analysis of the cytochrome c oxidase I (CO1) gene from selected populations of *D. polymorpha*. Values above the branch nodes represent the posterior probability (*p*) values derived from Bayesian inference analyses.

A scanning program was coded in C++ to search GenBank for *D. polymorpha polymorpha* DNA sequence files. After investigating 1226 individual submissions, the cytochrome c oxidase 1 (CO1) genetic marker was chosen due to its overrepresentation in population genetic studies relative to other markers for this species. This was likely due to a large number of DNA barcoding projects. Sequences that were neither verified nor linked to published research and those that could not be traced to a geographic locality were discarded. To confirm collection dates, sequences were cross-referenced to their corresponding publications and in cases where no collection date was specified, authors were contacted directly for confirmation. Based on the aforementioned parameters, a total of 25 CO1 sequences were acquired from Lake Superior and 58 from Europe (Figure 1(A), Table 1 and Online Supplementary Table). Sequences were aligned using the MUSCLE algorithm and edited by eye in Geneious ver. 10.1.3 (Kearse et al. 2012). This resulted in a 641-bp alignment with 151 variable sites.

**Table 1.** Dataset of verified cytochrome c oxidase 1 (CO1) sequences of the zebra mussel, *Dreissena polymorpha* obtained from the NCBI database, GenBank^a^ along with year(s) specimens were sampled.

Geographic locality	No. of sequences	Sampling year(s)
North America (Lake Superior)	24	2005/2006
Caspian Sea	8	2001
Turkey	6	2003
Italy	17	2006
Germany	7	2006
Ukraine	2	2000
Montenegro	3	2003–2009
Macedonia	2	2003/2004
France	5	2009
Hungary	4	1991
Croatia	1	2011

^a^ Accession numbers available in Supplementary table.

To investigate the strength of the population genetic signal, a Bayesian analysis was carried out in MrBayes ver. 3.2 (Ronquist et al. 2012) following the HKY nucleotide substitution model as determined by the Akaike Information Criterion (AICc index) in jModelTest ver 2.1 (Darriba et al. 2012). Bayesian analyses were initiated for 300 million generations in two separate runs, with 25% of trees discarded as burn-in and sample frequency set to 100. To determine haplotype distribution, sequences were collapsed into haplotypes and a parsimony network was constructed using TCS ver. 1.2.1 (Clement et al. 2000), with the fixed connection limit set to 95%. To estimate genetic differentiation, a hierarchical AMOVA (Analysis of Molecular Variance) along with a calculation of the analogue of Wrights Fixation index, ϕ_ST_ was conducted in Arelquin 3.5 (Excoffier and Lischer 2010).

Bayesian analysis showed strong population genetic signal with two major clades represented; a North American and European clade (Figure 1(B)). All haplotypes successfully connected with a 95% connection limit. A total of 22 unique haplotypes was recovered and a parsimony network showed significant geographic patterning (Online Supplementary Figure 1). All LS specimens formed a distinct haplogroup, which was extremely isolated from their European cohorts by more than 50 mutational steps. In contrast, within the European clade, parsimony networks showed a lack of any geographic patterning. For example, the haplotype EU2 was shared by individuals from nine geographically distinct regions. While Turkish zebra mussels were more related to European than North American individuals, they also clustered into a ‘mini’-haplogroup. The disjuncted haplotype distribution was corroborated by marked genetic differentiation between the two continents based on AMOVA results (ϕ_ST=_0.94, *p* < .05).

## Discussion

A combination of geographically patterned haplotype networks and significant AMOVA results revealed extremely limited gene flow which is in contrast to previous research on this species which found mixed haplotype networks and minimal to moderate levels of genetic differentiation between the Great Lakes and European populations (May et al. 2006 and references therein) One plausible explanation for the strong genetic differentiation observed is local adaptation. Aggregations of *D. polymorpha* and other dreissenids in Lake Superior are mainly limited to the Duluth Harbor which is regarded as the point source for *D. polymorpha*’s introduction to LS and also the sampling locality for where the archived sequences used in this study originated (Grigorovich et al. 2008). Unlike the rest of the Great Lakes, Lake Superior is interesting in that it possesses suboptimal conditions for *D. polymorpha* proliferation which includes extremely low temperatures coupled with low nutrient and mineral concentrations (especially Ca^2+^) (Grigorovich et al. 2003). These conditions could have acted as selection pressures and coupled with decreases in vector strength, may have driven the divergence of propagules that became established in the lake over time. To test this hypothesis, future studies incorporating morpho-genetic analyses and traditional garden experiments would be needed. Alternatively, there is the possibility that genetic drift may have influenced the high degree of structure observed. Drift, which has a more severe effect on mtDNA than nuclear DNA, could have been caused by repeated bottlenecks, a phenomenon that is common in the invasion process and which one would expect if populations are being continually introduced to habitats with sub-optimal conditions.

Sequence re-assembly and alignment was relatively straightforward. However, we did encounter incomplete records in some studies and, in such cases, it is likely that the handling editors and or reviewers failed to verify the accession numbers prior to final acceptance of the manuscript. For some records, source modifiers such as collection dates and or sampling localities were missing. In addition, most sequences could not be geo-referenced to GPS co-ordinates which limited the types of analyses that could be conducted (e.g. isolation by distance). Despite these challenges, the current study shows that informative results can be gleaned from sequence databases but improvements in the system will be needed before invasion biologists can reliably use them.

## Supplementary Material

Andrew_David_and_Kendall_Gardner_supplemental_content.zip
