# Supplementary figures and images for: Repurposing of archived CO1 sequence data reveals unusually high genetic structure between North American and European zebra mussels (Dreissena polymorpha)
Source: Mitochondrial DNA B Resour. 2017 Nov 25;2(2):853–5. doi: 10.1080/23802359.2017.1407713 (PMC7800893; doi:10.1080/23802359.2017.1407713)

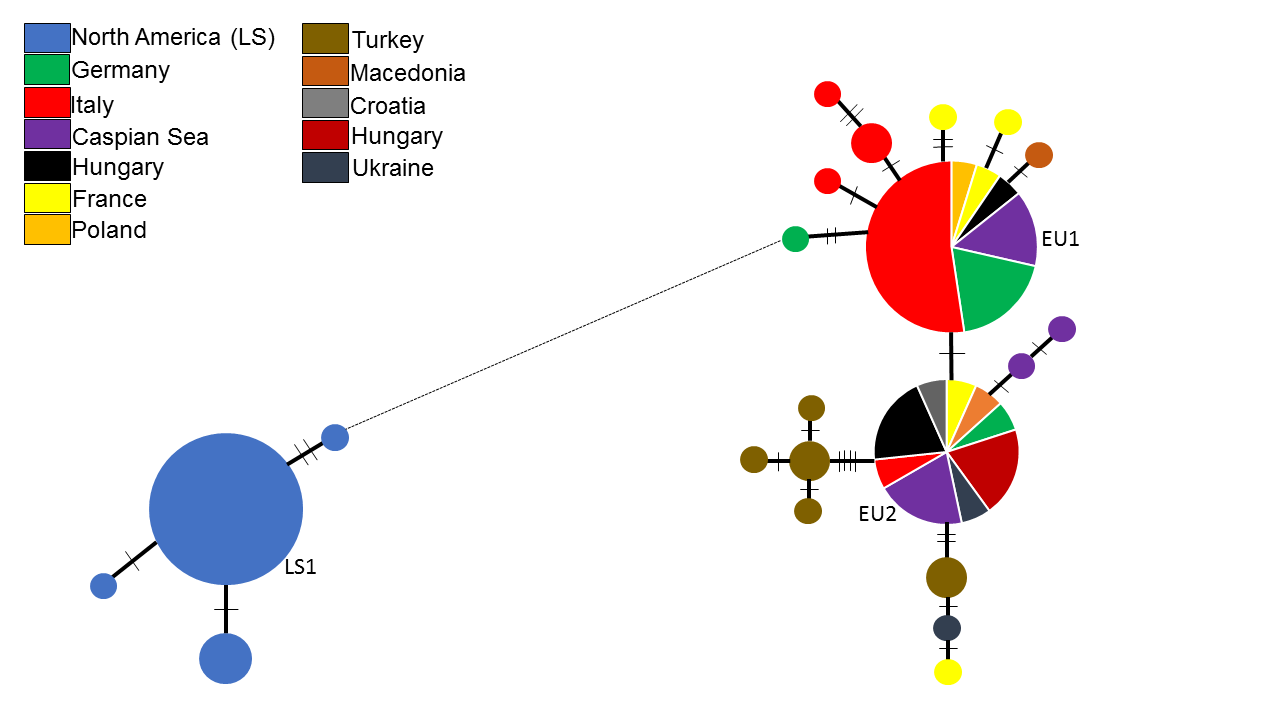

Supplement: Andrew_David_and_Kendall_Gardner_supplemental_content.zip [file TMDN_A_1407713_SM8719.zip › Supplementary_Figure_1.tif]
